# 1,3-Dimethyl-3-tetra­decyl-1*H*-1,5-benzodiazepine-2,4(3*H*,5*H*)-dione

**DOI:** 10.1107/S1600536811005782

**Published:** 2011-02-19

**Authors:** Rachid Dardouri, Fouad Ouazzani Chahdi, Natalie Saffon, El Mokhtar Essassi, Seik Weng Ng

**Affiliations:** a Laboratoire de Chimie Organique Hétérocyclique, Pôle de Compétences Pharmacochimie, Université Mohammed V-Agdal, B.P. 1014 Avenue Ibn Batout, Rabat, Morocco; b Service Commun Rayons-X FR2599, Université Paul Sabatier, Bâtiment 2R1, 118 route de Narbonne, Toulouse, France; c Department of Chemistry, University of Malaya, 50603 Kuala Lumpur, Malaysia

## Abstract

The seven-membered ring of the title compound, C_25_H_40_N_2_O_2_, adopts a boat-shaped conformation (with the C atoms of the fused-ring as the stern and the methine C atom as the prow). The tetra­decyl substituent occupies an equatorial position, with the tetra­dodecyl chain exhibibiting an an all-*trans* conformation.

## Related literature

For the crystal structure of the 12-bromo­dodecyl-substituted analog, see: Dardouri *et al.* (2010 ▶).
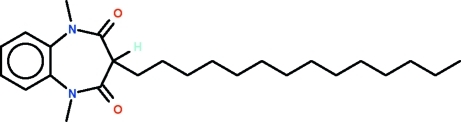


## Experimental

### 

#### Crystal data

C_25_H_40_N_2_O_2_
*M* *_r_* = 400.59
Monoclinic, 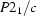

*a* = 8.1286 (1) Å
*b* = 33.5899 (5) Å
*c* = 9.4095 (2) Å
β = 114.640 (1)°
*V* = 2335.22 (7) Å^3^
*Z* = 4
Mo *K*α radiation
μ = 0.07 mm^−1^
*T* = 295 K
0.20 × 0.02 × 0.02 mm

#### Data collection

Bruker X8 APEXII diffractometer
29471 measured reflections
5359 independent reflections
3671 reflections with *I* > 2σ(*I*)
*R*_int_ = 0.044

#### Refinement

*R*[*F* ^2^ > 2σ(*F* ^2^)] = 0.047
*wR*(*F* ^2^) = 0.139
*S* = 1.03
5359 reflections
264 parameters
H-atom parameters constrained
Δρ_max_ = 0.23 e Å^−3^
Δρ_min_ = −0.19 e Å^−3^

### 

Data collection: *APEX2* (Bruker, 2008 ▶); cell refinement: *SAINT* (Bruker, 2008 ▶); data reduction: *SAINT*; program(s) used to solve structure: *SHELXS97* (Sheldrick, 2008 ▶); program(s) used to refine structure: *SHELXL97* (Sheldrick, 2008 ▶); molecular graphics: *X-SEED* (Barbour, 2001 ▶); software used to prepare material for publication: *publCIF* (Westrip, 2010 ▶).

## Supplementary Material

Crystal structure: contains datablocks global, I. DOI: 10.1107/S1600536811005782/bt5477sup1.cif

Structure factors: contains datablocks I. DOI: 10.1107/S1600536811005782/bt5477Isup2.hkl

Additional supplementary materials:  crystallographic information; 3D view; checkCIF report

## Figures and Tables

**Fig. 1.**
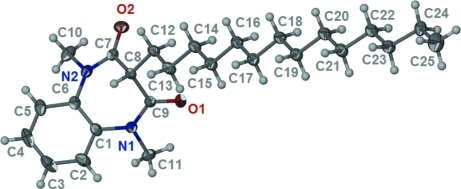
Anisotropic displacement ellipsoid plot (Barbour, 2001) of C25H40N2O2 at the 50% probability level; hydrogen atoms are drawn as arbitrary radius.

## supplementary crystallographic information

### Comment

The methylene part of 1,5-dimethyl-1,5-benzodiazepine-2,4-dione is relatively acidic, and one proton can be abstracted by using potassium *t*-butoxide; the resulting carbanion can undergo a nucleophlilic subsitution with a dibromoalkane to form 3-substituted derivatives. In a previous study, the compound was reacted with 1,12-dibromododecane to give the 12-bromododecyl substitued derivative (Dardouri *et al.*, 2010). The corresponding tetradecyl title compound (Scheme I, Fig. 1) was obtained by using 1-bromotetradecane.

### Experimental

To a solution of the potassium *t*-butoxide (0.42 g, 3.6 mmol) in DMF (15 ml) was added 1,5-dimethyl-1,5-benzodiazepine-2,4-dione (0.50 g, 2.4 mmol) and 1-bromotetradecane (0.78 ml, 2.88 mmol). Stirring was continued for 24 h. The reaction was monitored by thin layer ch romatography. The mixture was filtered and the solution evaporated to give colorless crystals.

### Refinement

H-atoms were placed in calculated positions (C—H 0.93–0.97 Å) and were included in the refinement in the riding model approximation, with *U*(H) set to 1.2–1.5*U*_eq_(C).

### Crystal data

**Table:**

C_25_H_40_N_2_O_2_	*F*(000) = 880
*M**_r_* = 400.59	*D*_x_ = 1.139 Mg m^−^^3^
Monoclinic, *P*2_1_/*c*	Mo *K*α radiation, λ = 0.71073 Å
Hall symbol: -P 2ybc	Cell parameters from 5173 reflections
*a* = 8.1286 (1) Å	θ = 2.5–26.9°
*b* = 33.5899 (5) Å	µ = 0.07 mm^−^^1^
*c* = 9.4095 (2) Å	*T* = 295 K
β = 114.640 (1)°	Plate, colorless
*V* = 2335.22 (7) Å^3^	0.20 × 0.02 × 0.02 mm
*Z* = 4	

### Data collection

**Table:**

Bruker X8 APEXII diffractometer	3671 reflections with *I* > 2σ(*I*)
Radiation source: fine-focus sealed tube	*R*_int_ = 0.044
graphite	θ_max_ = 27.5°, θ_min_ = 2.5°
φ and ω scans	*h* = −10→10
29471 measured reflections	*k* = −43→43
5359 independent reflections	*l* = −12→12

### Refinement

**Table:**

Refinement on *F*^2^	Primary atom site location: structure-invariant direct methods
Least-squares matrix: full	Secondary atom site location: difference Fourier map
*R*[*F*^2^ > 2σ(*F*^2^)] = 0.047	Hydrogen site location: inferred from neighbouring sites
*wR*(*F*^2^) = 0.139	H-atom parameters constrained
*S* = 1.03	*w* = 1/[σ^2^(*F*_o_^2^) + (0.0639*P*)^2^ + 0.5157*P*] where *P* = (*F*_o_^2^ + 2*F*_c_^2^)/3
5359 reflections	(Δ/σ)_max_ = 0.001
264 parameters	Δρ_max_ = 0.23 e Å^−^^3^
0 restraints	Δρ_min_ = −0.19 e Å^−^^3^

### Fractional atomic coordinates and isotropic or equivalent isotropic displacement parameters (Å^2^)

**Table:**

	*x*	*y*	*z*	*U*_iso_*/*U*_eq_	
O1	0.17583 (14)	0.70600 (3)	0.42788 (12)	0.0335 (3)	
O2	−0.24455 (15)	0.66528 (3)	0.46476 (13)	0.0383 (3)	
N1	−0.04415 (16)	0.72024 (4)	0.19047 (14)	0.0286 (3)	
N2	−0.36308 (16)	0.69006 (4)	0.21941 (14)	0.0280 (3)	
C1	−0.2029 (2)	0.70901 (4)	0.05774 (17)	0.0288 (3)	
C2	−0.2041 (3)	0.71320 (5)	−0.09017 (19)	0.0405 (4)	
H2	−0.1015	0.7227	−0.0994	0.049*	
C3	−0.3559 (3)	0.70346 (6)	−0.2228 (2)	0.0492 (5)	
H3	−0.3547	0.7059	−0.3208	0.059*	
C4	−0.5087 (3)	0.69014 (5)	−0.2098 (2)	0.0485 (5)	
H4	−0.6116	0.6839	−0.2992	0.058*	
C5	−0.5099 (2)	0.68592 (5)	−0.06424 (19)	0.0393 (4)	
H5	−0.6144	0.6770	−0.0567	0.047*	
C6	−0.3568 (2)	0.69484 (4)	0.07174 (17)	0.0281 (3)	
C7	−0.2317 (2)	0.67078 (4)	0.34230 (17)	0.0261 (3)	
C8	−0.06497 (19)	0.65811 (4)	0.31754 (16)	0.0240 (3)	
H8	−0.1044	0.6453	0.2150	0.029*	
C9	0.03579 (19)	0.69634 (4)	0.31786 (16)	0.0253 (3)	
C10	−0.5273 (2)	0.70170 (5)	0.2374 (2)	0.0408 (4)	
H10A	−0.4948	0.7121	0.3407	0.061*	
H10B	−0.6041	0.6789	0.2217	0.061*	
H10C	−0.5907	0.7218	0.1616	0.061*	
C11	0.0474 (2)	0.75737 (5)	0.1834 (2)	0.0390 (4)	
H11A	0.1248	0.7658	0.2875	0.059*	
H11B	−0.0413	0.7776	0.1330	0.059*	
H11C	0.1185	0.7530	0.1251	0.059*	
C12	0.0535 (2)	0.62912 (5)	0.44311 (17)	0.0282 (3)	
H12A	0.1026	0.6426	0.5433	0.034*	
H12B	−0.0207	0.6072	0.4499	0.034*	
C13	0.2087 (2)	0.61250 (5)	0.41109 (17)	0.0320 (4)	
H13A	0.2758	0.6345	0.3939	0.038*	
H13B	0.1594	0.5968	0.3160	0.038*	
C14	0.3380 (2)	0.58674 (5)	0.54351 (17)	0.0303 (3)	
H14A	0.3848	0.6023	0.6390	0.036*	
H14B	0.2711	0.5645	0.5590	0.036*	
C15	0.4961 (2)	0.57070 (5)	0.51467 (18)	0.0314 (4)	
H15A	0.5619	0.5929	0.4974	0.038*	
H15B	0.4496	0.5547	0.4203	0.038*	
C16	0.6259 (2)	0.54562 (5)	0.64913 (17)	0.0304 (3)	
H16A	0.5594	0.5237	0.6672	0.037*	
H16B	0.6732	0.5618	0.7431	0.037*	
C17	0.7836 (2)	0.52895 (5)	0.62100 (17)	0.0302 (3)	
H17A	0.8486	0.5508	0.6007	0.036*	
H17B	0.7365	0.5123	0.5285	0.036*	
C18	0.9155 (2)	0.50462 (5)	0.75784 (17)	0.0307 (3)	
H18A	0.9618	0.5213	0.8505	0.037*	
H18B	0.8505	0.4827	0.7777	0.037*	
C19	1.0743 (2)	0.48805 (5)	0.73090 (17)	0.0309 (3)	
H19A	1.1351	0.5098	0.7048	0.037*	
H19B	1.0286	0.4701	0.6422	0.037*	
C20	1.2114 (2)	0.46592 (5)	0.87169 (18)	0.0331 (4)	
H20A	1.2563	0.4839	0.9604	0.040*	
H20B	1.1503	0.4441	0.8975	0.040*	
C21	1.3712 (2)	0.44929 (5)	0.84705 (18)	0.0333 (4)	
H21A	1.4266	0.4706	0.8128	0.040*	
H21B	1.3276	0.4296	0.7643	0.040*	
C22	1.5143 (2)	0.43030 (5)	0.99273 (18)	0.0323 (4)	
H22A	1.4584	0.4092	1.0276	0.039*	
H22B	1.5587	0.4501	1.0750	0.039*	
C23	1.6742 (2)	0.41310 (5)	0.96896 (18)	0.0332 (4)	
H23A	1.6318	0.3914	0.8942	0.040*	
H23B	1.7234	0.4335	0.9248	0.040*	
C24	1.8235 (2)	0.39779 (5)	1.11869 (19)	0.0349 (4)	
H24A	1.7718	0.3792	1.1678	0.042*	
H24B	1.8733	0.4200	1.1898	0.042*	
C25	1.9760 (3)	0.37722 (6)	1.0943 (3)	0.0550 (5)	
H25A	2.0660	0.3685	1.1933	0.083*	
H25B	2.0295	0.3955	1.0474	0.083*	
H25C	1.9288	0.3547	1.0267	0.083*	

### Atomic displacement parameters (Å^2^)

**Table:**

	*U*^11^	*U*^22^	*U*^33^	*U*^12^	*U*^13^	*U*^23^
O1	0.0262 (6)	0.0391 (6)	0.0304 (6)	−0.0010 (5)	0.0069 (5)	0.0018 (5)
O2	0.0396 (7)	0.0461 (7)	0.0365 (6)	0.0029 (5)	0.0229 (5)	0.0055 (5)
N1	0.0229 (7)	0.0311 (7)	0.0305 (7)	0.0021 (5)	0.0099 (5)	0.0082 (5)
N2	0.0206 (6)	0.0278 (6)	0.0359 (7)	0.0023 (5)	0.0121 (5)	0.0007 (5)
C1	0.0275 (8)	0.0286 (8)	0.0271 (7)	0.0093 (6)	0.0084 (6)	0.0047 (6)
C2	0.0442 (10)	0.0446 (10)	0.0330 (9)	0.0161 (8)	0.0164 (8)	0.0131 (7)
C3	0.0654 (13)	0.0466 (11)	0.0275 (8)	0.0204 (10)	0.0113 (9)	0.0077 (8)
C4	0.0525 (12)	0.0354 (9)	0.0321 (9)	0.0075 (8)	−0.0075 (8)	−0.0010 (7)
C5	0.0333 (9)	0.0291 (8)	0.0401 (9)	0.0025 (7)	−0.0001 (7)	0.0024 (7)
C6	0.0271 (8)	0.0216 (7)	0.0307 (8)	0.0058 (6)	0.0071 (6)	0.0013 (6)
C7	0.0248 (8)	0.0240 (7)	0.0300 (7)	−0.0013 (6)	0.0119 (6)	−0.0012 (6)
C8	0.0242 (8)	0.0260 (7)	0.0213 (7)	0.0034 (6)	0.0090 (6)	0.0018 (6)
C9	0.0225 (8)	0.0300 (8)	0.0250 (7)	0.0041 (6)	0.0115 (6)	0.0013 (6)
C10	0.0275 (9)	0.0382 (9)	0.0611 (11)	0.0025 (7)	0.0227 (8)	0.0008 (8)
C11	0.0305 (9)	0.0395 (9)	0.0488 (10)	−0.0003 (7)	0.0182 (8)	0.0147 (8)
C12	0.0282 (8)	0.0300 (8)	0.0262 (7)	0.0061 (6)	0.0111 (6)	0.0068 (6)
C13	0.0350 (9)	0.0324 (8)	0.0286 (8)	0.0109 (7)	0.0133 (7)	0.0076 (6)
C14	0.0301 (8)	0.0313 (8)	0.0286 (8)	0.0078 (6)	0.0115 (7)	0.0062 (6)
C15	0.0300 (8)	0.0331 (8)	0.0303 (8)	0.0081 (6)	0.0117 (7)	0.0064 (6)
C16	0.0277 (8)	0.0339 (8)	0.0288 (8)	0.0060 (6)	0.0108 (7)	0.0053 (6)
C17	0.0279 (8)	0.0327 (8)	0.0289 (8)	0.0051 (6)	0.0108 (7)	0.0042 (6)
C18	0.0275 (8)	0.0328 (8)	0.0312 (8)	0.0064 (6)	0.0118 (7)	0.0074 (6)
C19	0.0296 (9)	0.0327 (8)	0.0296 (8)	0.0063 (7)	0.0114 (7)	0.0043 (6)
C20	0.0301 (9)	0.0368 (9)	0.0330 (8)	0.0081 (7)	0.0138 (7)	0.0071 (7)
C21	0.0312 (9)	0.0366 (9)	0.0308 (8)	0.0079 (7)	0.0117 (7)	0.0041 (7)
C22	0.0300 (9)	0.0345 (8)	0.0323 (8)	0.0069 (7)	0.0130 (7)	0.0046 (7)
C23	0.0319 (9)	0.0343 (8)	0.0345 (8)	0.0079 (7)	0.0149 (7)	0.0051 (7)
C24	0.0284 (8)	0.0312 (8)	0.0399 (9)	0.0024 (7)	0.0091 (7)	0.0006 (7)
C25	0.0388 (11)	0.0557 (12)	0.0656 (13)	0.0178 (9)	0.0169 (10)	0.0056 (10)

### Geometric parameters (Å, °)

**Table:**

O1—C9	1.2208 (17)	C14—H14A	0.9700
O2—C7	1.2139 (18)	C14—H14B	0.9700
N1—C9	1.3618 (18)	C15—C16	1.520 (2)
N1—C1	1.4228 (19)	C15—H15A	0.9700
N1—C11	1.468 (2)	C15—H15B	0.9700
N2—C7	1.3666 (19)	C16—C17	1.520 (2)
N2—C6	1.421 (2)	C16—H16A	0.9700
N2—C10	1.467 (2)	C16—H16B	0.9700
C1—C2	1.395 (2)	C17—C18	1.525 (2)
C1—C6	1.395 (2)	C17—H17A	0.9700
C2—C3	1.379 (3)	C17—H17B	0.9700
C2—H2	0.9300	C18—C19	1.521 (2)
C3—C4	1.373 (3)	C18—H18A	0.9700
C3—H3	0.9300	C18—H18B	0.9700
C4—C5	1.381 (3)	C19—C20	1.523 (2)
C4—H4	0.9300	C19—H19A	0.9700
C5—C6	1.396 (2)	C19—H19B	0.9700
C5—H5	0.9300	C20—C21	1.519 (2)
C7—C8	1.528 (2)	C20—H20A	0.9700
C8—C9	1.522 (2)	C20—H20B	0.9700
C8—C12	1.5242 (19)	C21—C22	1.520 (2)
C8—H8	0.9800	C21—H21A	0.9700
C10—H10A	0.9600	C21—H21B	0.9700
C10—H10B	0.9600	C22—C23	1.522 (2)
C10—H10C	0.9600	C22—H22A	0.9700
C11—H11A	0.9600	C22—H22B	0.9700
C11—H11B	0.9600	C23—C24	1.516 (2)
C11—H11C	0.9600	C23—H23A	0.9700
C12—C13	1.520 (2)	C23—H23B	0.9700
C12—H12A	0.9700	C24—C25	1.517 (2)
C12—H12B	0.9700	C24—H24A	0.9700
C13—C14	1.5210 (19)	C24—H24B	0.9700
C13—H13A	0.9700	C25—H25A	0.9600
C13—H13B	0.9700	C25—H25B	0.9600
C14—C15	1.519 (2)	C25—H25C	0.9600
			
C9—N1—C1	122.79 (13)	C14—C15—C16	113.18 (13)
C9—N1—C11	118.43 (13)	C14—C15—H15A	108.9
C1—N1—C11	118.49 (12)	C16—C15—H15A	108.9
C7—N2—C6	123.17 (12)	C14—C15—H15B	108.9
C7—N2—C10	117.17 (13)	C16—C15—H15B	108.9
C6—N2—C10	119.21 (13)	H15A—C15—H15B	107.8
C2—C1—C6	119.69 (15)	C15—C16—C17	113.74 (13)
C2—C1—N1	118.38 (15)	C15—C16—H16A	108.8
C6—C1—N1	121.92 (13)	C17—C16—H16A	108.8
C3—C2—C1	120.69 (18)	C15—C16—H16B	108.8
C3—C2—H2	119.7	C17—C16—H16B	108.8
C1—C2—H2	119.7	H16A—C16—H16B	107.7
C4—C3—C2	119.84 (17)	C16—C17—C18	113.44 (13)
C4—C3—H3	120.1	C16—C17—H17A	108.9
C2—C3—H3	120.1	C18—C17—H17A	108.9
C3—C4—C5	120.19 (16)	C16—C17—H17B	108.9
C3—C4—H4	119.9	C18—C17—H17B	108.9
C5—C4—H4	119.9	H17A—C17—H17B	107.7
C4—C5—C6	120.97 (18)	C19—C18—C17	113.73 (13)
C4—C5—H5	119.5	C19—C18—H18A	108.8
C6—C5—H5	119.5	C17—C18—H18A	108.8
C1—C6—C5	118.59 (15)	C19—C18—H18B	108.8
C1—C6—N2	122.00 (13)	C17—C18—H18B	108.8
C5—C6—N2	119.39 (15)	H18A—C18—H18B	107.7
O2—C7—N2	121.93 (14)	C18—C19—C20	113.43 (13)
O2—C7—C8	122.37 (13)	C18—C19—H19A	108.9
N2—C7—C8	115.66 (12)	C20—C19—H19A	108.9
C9—C8—C12	111.82 (12)	C18—C19—H19B	108.9
C9—C8—C7	105.98 (11)	C20—C19—H19B	108.9
C12—C8—C7	111.95 (12)	H19A—C19—H19B	107.7
C9—C8—H8	109.0	C21—C20—C19	114.14 (13)
C12—C8—H8	109.0	C21—C20—H20A	108.7
C7—C8—H8	109.0	C19—C20—H20A	108.7
O1—C9—N1	121.58 (14)	C21—C20—H20B	108.7
O1—C9—C8	122.46 (13)	C19—C20—H20B	108.7
N1—C9—C8	115.90 (12)	H20A—C20—H20B	107.6
N2—C10—H10A	109.5	C20—C21—C22	113.48 (13)
N2—C10—H10B	109.5	C20—C21—H21A	108.9
H10A—C10—H10B	109.5	C22—C21—H21A	108.9
N2—C10—H10C	109.5	C20—C21—H21B	108.9
H10A—C10—H10C	109.5	C22—C21—H21B	108.9
H10B—C10—H10C	109.5	H21A—C21—H21B	107.7
N1—C11—H11A	109.5	C21—C22—C23	113.96 (13)
N1—C11—H11B	109.5	C21—C22—H22A	108.8
H11A—C11—H11B	109.5	C23—C22—H22A	108.8
N1—C11—H11C	109.5	C21—C22—H22B	108.8
H11A—C11—H11C	109.5	C23—C22—H22B	108.8
H11B—C11—H11C	109.5	H22A—C22—H22B	107.7
C13—C12—C8	112.92 (12)	C24—C23—C22	113.22 (13)
C13—C12—H12A	109.0	C24—C23—H23A	108.9
C8—C12—H12A	109.0	C22—C23—H23A	108.9
C13—C12—H12B	109.0	C24—C23—H23B	108.9
C8—C12—H12B	109.0	C22—C23—H23B	108.9
H12A—C12—H12B	107.8	H23A—C23—H23B	107.7
C12—C13—C14	113.18 (12)	C23—C24—C25	113.69 (15)
C12—C13—H13A	108.9	C23—C24—H24A	108.8
C14—C13—H13A	108.9	C25—C24—H24A	108.8
C12—C13—H13B	108.9	C23—C24—H24B	108.8
C14—C13—H13B	108.9	C25—C24—H24B	108.8
H13A—C13—H13B	107.8	H24A—C24—H24B	107.7
C15—C14—C13	113.74 (12)	C24—C25—H25A	109.5
C15—C14—H14A	108.8	C24—C25—H25B	109.5
C13—C14—H14A	108.8	H25A—C25—H25B	109.5
C15—C14—H14B	108.8	C24—C25—H25C	109.5
C13—C14—H14B	108.8	H25A—C25—H25C	109.5
H14A—C14—H14B	107.7	H25B—C25—H25C	109.5
			
C9—N1—C1—C2	−130.73 (16)	O2—C7—C8—C12	15.1 (2)
C11—N1—C1—C2	43.0 (2)	N2—C7—C8—C12	−167.19 (12)
C9—N1—C1—C6	50.6 (2)	C1—N1—C9—O1	177.20 (14)
C11—N1—C1—C6	−135.64 (15)	C11—N1—C9—O1	3.4 (2)
C6—C1—C2—C3	0.0 (2)	C1—N1—C9—C8	−5.7 (2)
N1—C1—C2—C3	−178.74 (15)	C11—N1—C9—C8	−179.47 (13)
C1—C2—C3—C4	1.1 (3)	C12—C8—C9—O1	−16.4 (2)
C2—C3—C4—C5	−0.9 (3)	C7—C8—C9—O1	105.85 (15)
C3—C4—C5—C6	−0.4 (3)	C12—C8—C9—N1	166.53 (12)
C2—C1—C6—C5	−1.2 (2)	C7—C8—C9—N1	−71.22 (15)
N1—C1—C6—C5	177.45 (13)	C9—C8—C12—C13	−67.67 (16)
C2—C1—C6—N2	−179.89 (14)	C7—C8—C12—C13	173.57 (13)
N1—C1—C6—N2	−1.2 (2)	C8—C12—C13—C14	174.38 (13)
C4—C5—C6—C1	1.4 (2)	C12—C13—C14—C15	−178.69 (14)
C4—C5—C6—N2	−179.87 (14)	C13—C14—C15—C16	178.98 (14)
C7—N2—C6—C1	−49.2 (2)	C14—C15—C16—C17	179.30 (14)
C10—N2—C6—C1	138.72 (15)	C15—C16—C17—C18	178.70 (13)
C7—N2—C6—C5	132.13 (15)	C16—C17—C18—C19	−179.64 (13)
C10—N2—C6—C5	−39.9 (2)	C17—C18—C19—C20	176.52 (13)
C6—N2—C7—O2	−176.13 (14)	C18—C19—C20—C21	−179.80 (14)
C10—N2—C7—O2	−3.9 (2)	C19—C20—C21—C22	175.23 (14)
C6—N2—C7—C8	6.2 (2)	C20—C21—C22—C23	179.33 (14)
C10—N2—C7—C8	178.37 (13)	C21—C22—C23—C24	174.57 (14)
O2—C7—C8—C9	−107.07 (15)	C22—C23—C24—C25	174.79 (15)
N2—C7—C8—C9	70.64 (15)		
